# Guidelines for planning and conducting high-quality research and testing on animals

**DOI:** 10.1186/s42826-020-00054-0

**Published:** 2020-07-10

**Authors:** Adrian J. Smith

**Affiliations:** Norecopa, P.O. Box 750 Sentrum, 0106 Oslo, Norway

**Keywords:** Guidelines, Planning, Reporting, Animal, Research, Testing, PREPARE, Validity, Reproducibility

## Abstract

There are important scientific, legal and ethical reasons for optimising the quality of animal research and testing. Concerns about the reproducibility and translatability of animal studies are now being voiced not only by those opposed to animal use, but also by scientists themselves.

Many of the attempts to improve reproducibility have, until recently, focused on ways in which the reporting of animal studies can be improved. Many reporting guidelines have been written. Better reporting cannot, however, improve the quality of work that has already been carried out - for this purpose better planning is required.

Planning animal studies should involve close collaboration with the animal facility where the work is to be performed, from as early a stage as possible. In this way, weaknesses in the protocol will be detected and changes can be made before it is too late. Improved planning must focus on more than the “mathematical” elements of experimental design such as randomisation, blinding and statistical methods. This should include focus on practical details such as the standard of the facility, any need for education and training, and all the factors which can improve animal welfare.

The PREPARE (*Planning Research and Experimental Procedures on Animals: Recommendations for Excellence*) checklist was developed to help scientists be more aware of all the issues which may affect their experiments. The checklist is supported by comprehensive webpages containing more information, with links to the latest resources that have been developed for each topic on the list.

## Introduction

There is now widespread international acceptance for the 3R-concept (*Replacement, Reduction, Refinement* [1]) when planning research or testing which may involve the use of animals or animal tissue:

- Replacement where possible with non-animal methods
- Reduction of the number of animals to the minimum which achieves a valid result, and
- Refinement of the care and use of those animals which must be used, to maximise animal welfare and data quality.

The three Rs are now part of animal research legislation in many countries [2]. In Europe, the European Union Directive 2010/63 explicitly states that Replacement is the ultimate aim [3]. An assessment of the need to use animals at all must therefore be the first stage of the process when planning preclinical research or testing. The large range of alternatives now available is beyond the scope of this paper, but there are many sources of information of this topic (e.g. [4]).

If animal use is unavoidable, attention must be paid to a long list of known variables which may affect the data collected from them. Unlike test-tube ingredients, animals are complex individuals, differing in their genetic make-up, microbial composition, and behavioural responses to their environment and procedures to which they are subjected. Again, a review of all these factors is beyond the scope of this paper, but information on the effects of these variables is available (e.g. [5]).

In addition to the legal and scientific incentives, there are good ethical reasons for aiming for the highest possible quality of animal-based research and testing. This is particularly important to remember within basic research in academia, where scientists may be rewarded for the publication of new knowledge rather than for the application of their research results.

In most cases, animal research and testing is performed to learn more about another species, usually humans, rather than to shed more light on the species being used as a model. This work must, therefore, be valid, robust and translatable. As Ritskes-Hoitinga & Wever [6] remarked: ‘we need a cultural change in which researchers are rewarded for producing valid and reproducible results that are relevant to patients, and for doing justice to the animals being used’. Ensuring translatability is difficult enough in itself [7], and it is totally dependent upon well-planned studies.

Quality does not come automatically: it necessitates detailed planning from day one, to take into account the effects of the internal and external parameters which affect the animals’ response to a procedure. In addition, the animal facility must have a large number of routine procedures in place, both to maintain the stability of the environment and to tackle any emergencies which may arise. Many scientists who do not work on a regular basis within an animal facility are probably unaware of the number and subtlety of many of these factors. Input from the facility’s veterinary staff will be central to this process.

Guidelines for planning and conducting animal-based studies help both scientists and animal facilities to discuss the issues mentioned above at an early stage, while it is still possible to make improvements in the protocol. Scientists may need to be reminded that the greatest source of variation is likely to come from the animals themselves, rather than from their treatments. Scientists may assume that the facility is dealing with these issues, but this is not always the case. The classic studies by Crabbe and coworkers, who set up standardised behavioural tests on inbred mouse strains in different laboratories simultaneously, showed how unforeseen variables can lead to significant differences in results [8, 9].

Fortunately, the need for detailed planning guidelines is becoming clearer, because the quality of animal experiments is now increasingly being criticised, not just by opponents of animal research but also by scientists themselves (e.g. [10–14]). The use of strong words such as ‘research waste’ and ‘false results’ (e.g. [15, 16]) is becoming commonplace.

Unfortunately, initiatives to solve the reproducibility crisis tend often to focus on just two of the issues: the more “mathematical” elements of experimental design, and better reporting (e.g. [17]). These issues are of course important, and include the following items, among others:

1. Publication bias (reporting only positive results)
2. Low statistical power
3. *P*-value hacking (manipulating data to obtain statistical significance)
4. HARKing *(Hypothesising After the Results are Known)*
5. Lack of randomisation and blinding

Norecopa has made a collection of literature references about these concerns [18].

However, those familiar with the workings of an animal facility can add many additional and important issues to this list, which may be less conspicuous but which are equally critical to the validity of an experiment. These may be grouped into:

1. Artefacts caused by internal factors such as genetic diversity and subclinical infections
2. Artefacts caused by external effects such as transport, cage conditions, re-grouping of animals, food deprivation and the procedure itself
3. The need for contingency plans to reduce or avoid these and other risks in the facility

## Reporting does not improve the standard of experiments

Good reporting is of course important, to allow readers to evaluate the scientific quality of the publication and the strength of the conclusions drawn by the authors. Insistence on better reporting is not new. When Laboratory Animal Science as we know it today was under development in the second half of the last century, focus was placed at an early stage on the low standard of reporting in the scientific literature. In a classic paper, Jane Smith and colleagues [19] examined the descriptions of laboratory animals, and the procedures for which they were used, in 149 scientific papers published in 8 major journals from 1990 to 1991. The percentages of papers not reporting basic details about the animals were alarmingly high (e.g. sex: 28%; age: 52%; weight: 71%; source: 53%), and 30% of the papers did not mention how many animals were used. The percentages were even higher for environmental factors such as room temperature (72%), photoperiod (72%), relative humidity (89%) and the number of animals per cage (73%).

Many reporting guidelines have been written since the 1980s, to encourage improvements. These include both general guidance (e.g. [20–24]) and guidelines written for specific types of experiment (e.g. [25–28]).

It is vitally important to remember that better reporting of an experiment which has already been performed cannot improve the quality of that experiment. A good salesman may manage to sell more burnt cakes if he describes them well (and if he is a good psychologist), but they will still be burnt and they will not taste better. To improve a cake, one must go back to the kitchen and modify the ingredients and/or the baking conditions. In the case of animal studies, just as in the kitchen, the quality of the result is dependent upon planning and conducting, not reporting.

This has been well demonstrated by the way in which the ARRIVE (*Animal Research: Reporting of* In Vivo *Experiments*) guidelines for reporting animal experiments [23] have been received and implemented. A new version of ARRIVE was developed in 2019 [29], because, as the authors point out, despite endorsement by more than a thousand journals, only a small number of these journals actively enforce compliance. Indeed, a Swiss study revealed that 51% of researchers using journals that had endorsed ARRIVE had even never heard of them [30]. The authors of ARRIVE concluded that most journals are unlikely to be able to provide the resources needed to ensure compliance with all the items on the original checklist. The new version of the ARRIVE guidelines has a shorter checklist of ‘essential’ items, to try and increase compliance. This situation demonstrates clearly how important the *planning* stage is for the quality of scientific papers.

Scientists should contact the animal facility as soon as they have concrete plans of conducting animal studies. Collaboration between scientists and facility staff will be needed to discuss all stages of the study, up to and including the end of the study which involves depopulation, decontamination and waste disposal. An essential part of this process is attention to the needs of the facility staff. This includes, among other things, their education and training, personal protection, their workload, and means of ensuring adequate staffing levels at all times during the study.

## Preparation for preclinical studies: a modern definition of the 3Rs

The concept of the three Rs *(Replacement, Reduction and Refinement)* developed by Russell and Burch over 60 years ago [1] was written in an era when the most pressing need was to reduce the inhumanity of animal experiments. Technology at that time did not offer the same potential to replace such experiments as is available today - neither was there so much focus on reducing animal numbers by more sophisticated experimental design.

So today, preparation for robust, valid and humane preclinical studies should go beyond a mere search for more humane methods, using more contemporary definitions of the three Rs [31]:

- Replacement is not just the use of methods which achieve a given purpose without procedures on animals, but also about total avoidance of animal use (Non-Animal Models, NAMs) by innovative approaches to scientific problems, for example by studies directly on human tissue
- Reduction is about obtaining comparable information from fewer animals, or for obtaining more information from the same number of animals. Today, reduction also focuses on optimalisation of experimental design so that *experiments are robust and reproducible*
- Refinement methods minimise pain, suffering or distress, but also improve animals’ well-being, since modern research demonstrates that this affects the quality of the data collected from the animals. Modern technology can be harnessed to refine the methods and equipment we use on animals.

Animals that are in harmony with their surroundings will provide more reliable scientific data in an experiment, because the parameters measured will reflect the treatment they are given, rather than being affected by stress. It is indeed true that ‘happy animals give better science’ [32, 33].

For these reasons, scientists must be given comprehensive guidelines for planning any experiments which may involve the use of animals, or material taken from animals.

## The PREPARE guidelines

Based on the authors’ experiences over the past 30 years in designing and supervising animal experiments, comprehensive guidelines for planning animal studies have been constructed, called PREPARE (*Planning Research and Experimental Procedures on Animals: Recommendations for Excellence*) [34].

**Fig. 1:**
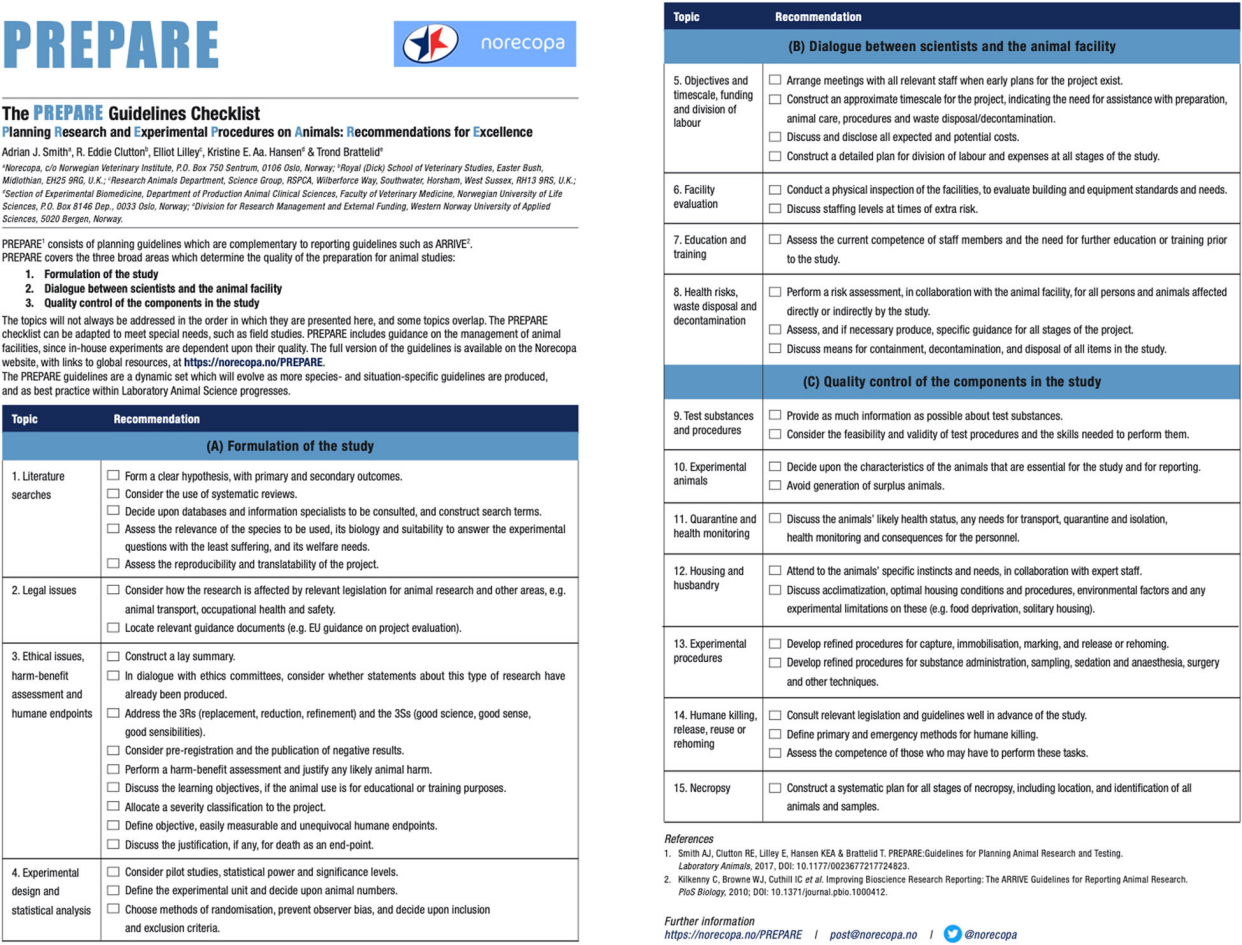
The PREPARE checklist (available at https://norecopa.no/PREPARE/prepare-checklist). From Smith, AJ, Clutton, RE, Lilley, E, Hansen KEAa, Brattelid, T. PREPARE: Guidelines for planning animal research and testing. Laboratory Animals, 2018;52:135–141. DOI: 10.1177/0023677217724823*.* Published under Open Access, Creative Commons licence CC BY-NC 4.0

PREPARE contains a checklist, which serves as a reminder of items that should be considered before and during the study, see Fig. 1. This checklist is available in over 20 languages.

Many of these items will need their own sets of checklists or standard operating procedures, in the same way that pilots, however experienced, use many checklists, even on routine flights, before, during and after the flight. Many of these checklists will be produced by the animal facility itself. Scientists should, however, check that these are in place, and discuss their contents with the facility.

Importantly, and unlike many reporting guidelines, the PREPARE checklist is supported by a website which provides more information on each of the 15 main topics on the checklist (https://norecopa.no/PREPARE). The website gives more complete guidance in the form of text and links to quality guidelines and scientific papers. This website is continually updated as new knowledge develops.

The PREPARE guidelines contain, of course, many of the elements found in reporting guidelines. However, PREPARE contains additional material about issues that can have dramatic effects on the scientific validity of the research, as well as on health and safety, and animal welfare.

## Contingency plans and resources

Human nature is such that we tend to believe that accidents only happen to others. If this belief is followed in an animal facility, it will not only put the outcome of a scientific study at risk, but it will also endanger the health and lives of both the animals and personnel who are directly or indirectly involved in the study. As indicated above, it is important to ensure the quality of the whole process from obtaining the animals to disposal of waste and decontamination after the study.

A competent animal facility is one that *“hopes for the best but is prepared for the worst”*. Facilities with comprehensive and realistic contingency plans will be well placed to tackle disasters, including lockdown situations in connection with a pandemic. There are many resources available that describe the general principles involved, but these must be tailored to the local conditions at each facility. Building a contingency plan from scratch is a time-consuming affair, but it is an excellent insurance policy for the day when a threatening situation arises. Those lacking such a plan should begin with a risk assessment of the facility and its activities, and start by writing contingency plans for the most important of these scenarios.

In its simplest terms, risk assessment is the consequence of a threat multiplied by the likelihood of it occurring. The consequences of the threat include the level of tolerance of the event occurring, which may be anything from “totally unacceptable” to “acceptable within certain limits”. Assessments should be performed at several levels, since threats and their consequences may differ, depending upon where and when they occur, for example:

- at the facility level (e.g. the consequences of flooding or fire)
- at the room level (e.g. the consequences of power outages to vital equipment)
- in connection with specific types of research (e.g. risk of human infection)

It is wise to construct a contingency plan based upon the assumption that ‘what can go wrong will go wrong at some time’ [35], and that this will happen when it is least convenient, for example during public holidays when staffing levels may be low.

Clearly, both the design of animal studies and the production of contingency plans must involve close collaboration between management, scientists and technical staff, including external suppliers of equipment and services.

The Covid-19 pandemic has demonstrated the importance of being adequately prepared. Animal facilities have had to quickly write contingency plans to tackle situations which were barely imaginable before the outbreak. This work has demanded enormous time and energy, at the expense of conducting research, and it has left many facilities with the unpleasant task of having to euthanise large numbers of healthy animals. Clearly, it is easier to tackle these situations, however improbable they may seem, if the majority of the issues which may arise have already been discussed, and plans made to tackle them.

At the time of writing, some specific advice on contingency plans for the Covid-19 pandemic is beginning to emerge, and existing advice on disaster planning is being re-examined (see [36]).

## Collaboration between scientists and animal care staff

There are many good reasons for early and close collaboration between scientists and the staff at the animal facility where they hope to carry out the work. This collaboration should include dialogue with the animal carers and technicians, not just with the managers. Some of the reasons include the following:

- the staff have a moral right to know what will happen to animals in their care.
- they will be more motivated to look for ways of refining the study. This will improve both animal welfare and the scientific quality, including reliability of the data being collected from the animals.
- the animal care staff know the possibilities, and the limitations, of the animal facility best. They are less likely to play limitations down for fear of the study being transferred to another facility.
- they often possess a large range of practical skills and are good at lateral thinking from one study to another - they may be able to suggest a refinement which they have already seen in another species.
- they know the animals best
- the animals know them best
- lack of involvement of the animal care staff creates anxiety, depression and opposition to animal research, as well as limiting creativity which might improve the experiments

A mutually respectful dialogue between technical and academic staff will also help resolve issues quickly which may otherwise cause disagreements later, such as the division of labour and responsibilities all stages of the study. It will also help to avoid the loss of important data due to misunderstandings about who was to collect it.

## Culture of care and challenge

To facilitate this dialogue, steps should be taken to foster a culture of care among all members of the staff and research teams. This is actively encouraged in European legislation [3]. Animal research will inevitably, from time to time, involve studies where sentient creatures exhibit pain, suffering and distress. It is therefore vital to consider the mental health of those caring for these animals or observing this, to avoid compassion fatigue. In Europe, an International Culture of Care network has been established, to share experiences in implementing such a culture [37].

Closely related to a culture of care is the concept of a Culture of Challenge [38]. This is all about ‘looking for the acceptable, rather than choosing the accepted’. Comments such as “we have always done it that way” or “we do it as often as necessary” should automatically start a discussion about how to change these habits.

## Conclusion

It can be hoped that the current focus on poor reproducibility in animal studies can be turned into an initiative to ensure better planning of all stages, rather than focusing on improving reporting. Otherwise, we are in danger of wasting time, discussing the quality of the lock on the door of the stable from which the horse has already escaped [39].

## Data Availability

This review paper does not include original data. Figure 1 is from a previous paper, published under Open Access, Creative Commons licence CC BY-NC 4.0.
